# An Online Experiment During COVID-19: Testing the Influences of Autonomy Support Toward Emotions and Academic Persistence

**DOI:** 10.3389/fpsyg.2021.747209

**Published:** 2021-10-11

**Authors:** Yurou Wang, Jihong Zhang, Halim Lee

**Affiliations:** ^1^Department of Educational Studies in Psychology, Research Methodology, and Counseling, The University of Alabama, Tuscaloosa, AL, United States; ^2^Department of Psychological and Quantitative Foundations, The University of Iowa, Iowa City, IA, United States

**Keywords:** academic persistence, emotion, autonomy support, online learning, joy, anxiety

## Abstract

Students’ academic persistence is a critical component of effective online learning. Promoting students’ academic persistence could potentially alleviate learning loss or drop-out, especially during challenging time like the COVID-19 pandemic. Previous research indicated that different emotions and autonomy support could all influence students’ academic persistence. However, few studies examined the multidimensionality of persistence using an experimental design with students’ real-time emotions. Using an experimental design and the Contain Intelligent Facial Expression Recognition System (CIFERS), this research explored the dynamic associations among real-time emotions (joy and anxiety), autonomy support (having choice and no choice), self-perceived persistence, self-reliance persistence, and help-seeking persistence. 177 college students participated in this study online via Zoom during COVID-19 university closure. The results revealed that having choice and high intensity of joy could promote students’ self-reliance persistence, but not help-seeking persistence. Interestingly, students who perceived themselves as more persistent experienced more joy during experiment. The theoretical and practical implications on facilitating students’ academic persistence were discussed.

## Introduction

A question educators and researchers frequently ask is how to encourage students to learn persistently, especially in online settings (e.g., Vansteenkiste et al., 2004; Jung and Lee, 2018). Under the COVID-19 pandemic, cultivating students’ learning persistence is more critical than ever in order to prevent learning loss and drop-out induced by school closures (Bao, 2020, 115; Dorn et al., 2020, 2-3). Despite learning loss, COVID-19 also raised mental health challenges, such as stress and anxiety, for college students (Odriozola-González et al., 2020; Charles et al., 2021). It is crucial to understand how to facilitate learning persistence while students are under stress and anxiety when going through challenging life incidences like the COVID-19 pandemic. Would autonomy support still effectively promote students’ learning persistence and positive emotions in terms of online learning experience during COVID, as suggested by self-determination theory (Deci and Ryan, 2000)? How do different kinds of emotions constrain or elicit academic persistence? The purposes of this study are (a) provide a better understanding of academic persistence; (b) explore the associations among emotions, choice (as the indicator of autonomy support), and academic persistence. Specifically, this study used an experimental design to answer the questions: How do different emotions (joy and anxiety) and with/without choice influence students’ academic persistence?

## Theoretical Background

Previous literature suggested the potential association among academic persistence, emotions, and autonomy support (e.g., Bonneville-Roussy et al., 2013; O’Neill and Thomson, 2013). The conceptualization of academic persistence and how emotion and autonomy influence academic persistence will be reviewed in this section.

### Academic Persistence

It is still hard to define academic persistence, as consensus has not been made concerning the definition of persistence. There are two major controversies: (1) trait vs. state; (2) help-seeking behavior. Early research defined academic persistence as the consistent investment in learning despite obstacles, difficulties, failures, and situations (Zimmerman and Risemberg, 1997). However, recent research argued that persistence is more complex (Roland et al., 2018). It can vary from person to person, depending on the situation and one’s personal preference. The early definition took the trait-dependent view and suggested that persistence is a stable trait, which means that a persistent person will struggle through hardship to achieve their goals across various domains and settings (Sellman et al., 1997; Sommer and Baumeister, 2002). However, the later definition took the state-dependent view that persistence is no more than a state, so people who persist in one context might not persist in a different situation (Baker et al., 2008; Hershkovitz and Nachmias, 2009).

The second controversy is around help-seeking behavior. Even though help-seeking behaviors could help students continue on a challenge or difficult task (Jackson et al., 2003; Terrell et al., 2015), most of the time, persistence was seen as a self-reliance physical and mental process. Some researchers viewed help-seeking behavior as a sign of weakness (Tyssen et al., 2004).

This research would like to propose a new definition for academic persistence to address the conceptual issues stated above. As discussed, previous research had debates around trait vs. state and whether help-seeking is a form of persistence. This research would like to move away from trait vs. state debate and trade help-seeking as a form of persistence because student overthrows their psychological barrier of being seen weak and tries to achieve their academic goals by asking for help. The current study defined academic persistence as an individual’s self-perceived and actual willpower and behaviors (may vary in different situations) to overcome obstacles, difficulties, and failures by oneself or by seeking help from others to achieve learning goals. This definition contains three types of persistence: self-perceived (trait), self-reliance (state), and help-seeking persistence (state). Self-perceived persistence is how a person thinks he or she will behave when facing difficulties and obstacles. The latter two types of persistence are individual’s actual reactions when facing difficulties or challenges in learning. Self-reliance persistence means a person continues to work hard on the problem by oneself. Help-seeking persistence is defined as a person seeks help from others to overcome obstacles and complete an arduous task.

Persistence is not only hard to define, it is also extremely hard to measure and quantify because of its multidimensionality and dynamic nature. Scholars used self-report scales (Renaud-Dubé et al., 2015) and the amount of time invested (Pelletier et al., 2001; Jõesaar et al., 2011) measure persistence. However, experimental studies of persistence are limited and often do not reflect real-world problem-solving scenarios. More importantly, few experimental studies have captured the multidimensionality of persistence. In this study, a self-reported scale and experimental design are implemented in order to capture the three aspects of persistence. The measurement of academic persistence will be specified in the “Materials and Methods” section.

Besides the calling for a better understanding of academic persistence, it is also essential to understand what supports students’ academic persistence. Whether persistence could depend on (a) students’ emotions (Tulis and Ainley, 2011); (b) autonomy support (Pelletier et al., 2001). The following section will review the literature on emotion and autonomy to explain how these two factors would influence academic persistence.

### Emotion

Emotions in this study were defined as the various emotions directly induced by learning activities and learning outcomes. Traditionally, researchers study cognition and emotion separately, and emotions have not been studied intensively in education before the 1990s (Pekrun, 2019). The recent 20 years sees a rise in studies of emotion in education, as emotions were discovered to activate and deactivate cognition and metacognition processes (e.g., persistence) related to learning (Artino and Jones, 2012; King and Areepattamannil, 2014; Ramirez-Arellano et al., 2019).

Emerging literature addresses the importance of emotions in the online learning context (e.g., Feidakis et al., 2014; D’Errico et al., 2016). O’Regan (2013) concluded that emotions played a central role in students’ lived online learning experience through interviews with eleven students. Both anxiety and excitement were stood out in students’ discussion of the online learning experience. D’Errico et al. (2018) also detected and classified 11 cognitive emotions students showed in video-lecture and chat with teachers. Parlangeli et al. (2012) argued that, within online learning, cognitive emotions were crucial, but social emotions also needed attention. Most of the studies concerning emotions in an online context aimed to address the importance of emotions or identify the types of emotions students demonstrated, but not much research explored the association of these emotions and students’ academic persistence.

However, in the traditional face-to-face learning context, there was burgeoning consciousness of the significant role of emotions in students’ academic performance (Tulis and Fulmer, 2013). Anxiety, especially test and math anxiety, was studied massively as a predictor for academic performance (Cassady and Johnson, 2002; Zeidner, 2014; Putwain et al., 2016). Anxiety is the outcome of negative (unpleasant) emotions like anger and frustration, and most of the time associated with academic performance negatively (Chapell et al., 2005; Karatas et al., 2013). There is also joy, which is seen as the outcome of enjoyment. Such joy of learning deepens the learning process and promotes academic achievement (Goetz et al., 2008; Villavicencio and Bernardo, 2013; Putwain et al., 2016).

Not only associated with academic performance, joy and anxiety also potentially related to academic persistence. Students who experience positive emotion (joy) would perceive themselves have enough ability or resources to achieve their goal. On the other hand, students with unpleasant emotions (anxiety) would be frustrated by the current situation and avoiding continuing their goals (Linnenbrink and Pintrich, 2002; Ainley et al., 2005; Tulis and Ainley, 2011). Aforementioned research suggested a potential association between these two emotions and students’ persistence, so the joy of solving specific problems or the anxiety activated by failures or challenging tasks would be this study’s focused emotions.

Most previous studies used questionnaires to measure emotions, for instance, the Achievement Emotions Questionnaire (AEQ; Pekrun et al., 2011) and the Epistemically-Related Emotion Scale (EES; Pekrun et al., 2017). However, the research mentioned above, and the self-reported questionnaires have several limitations. Firstly, they did not provide the real-time emotional status during students’ problem-solving process. Secondly, self-reported data has widely acknowledged drawbacks: for instance, participants would conceal their real opinion, and discrepancies might exist between how people behave and how people think they would behave (Sallis and Saelens, 2000; Subar et al., 2015). Thirdly, self-reported data does not provide observed valence and activation of synchronous emotions during task completion, but these characteristic of synchronous emotions are essential for learning (Pekrun, 2006).

To address these limitations, this study incorporated the Contain Intelligent Facial Expression Recognition System (CIFERS) to measure two dimensions of emotions: valence (positive or negative) and activation (activating or deactivating) (Pekrun, 2000, 2006). CIFERS could track students’ macro- and micro-facial expressions as indicators for different emotions (both positive and negative). It could also provide information on real-time emotion change, emotion intensity (activating and deactivating), as well as the specific time an emotion occurs. Before elucidating more on CIFERS in the “Materials and Methods” section, certain suspicion must be squelched: why use a facial expression as an emotion indicator and whether this approach is accurate? The implication of facial expression in emotion studies was presented below to answer these two questions.

### Facial Expression and Emotion

Facial expressions have long been used to indicate emotions and stayed central in emotion studies (Tomkins and McCarter, 1964; Russell, 1994; Ruba and Repacholi, 2020). The accuracy of using facial expressions to identify emotions has been justified through many ways, for instance, self-report instruments (Matsumoto, 1987; Matsumoto et al., 2000) and facial coding systems (Ekman et al., 1980; Clark et al., 2020; Rosenberg and Ekman, 2020). There is debate around the universality of facial expression. Early research discovered that even people in an isolated tribe in New Guinea shared the same emotional interpretation of facial expression (Izard, 1992). This finding was later replicated by Matsumoto (1992) and Ekman (1994). Other researchers questioned such findings. For instance, Jack et al. (2009, 2012) argued that facial expressions are not universal. However, they can only prove that the intensity of emotions and degree of the movement of people’s faces are different. More importantly, the differences they identified did not exist in facial expression but in how people use their own cultural understandings to interpret facial expressions. In this paper, we believed that facial expressions, both macro and micro facial expressions, are shared by different cultures; only the intensity and interpretation might be different from culture to culture (Ekman and Friesen, 2003; Cowen and Keltner, 2020).

The CIFERS equipment adopted in this research was established based on Ekman and Rosenberg (1997) and Rosenberg and Ekman (2020) facial expression theory and facial coding systems, which divided the face into 47 units. With CIFERS, the facial movement could be obtained within 50 ms. CIFERS’s basic mechanism is out of the scope of this research, but more information could be found in Supplementary Appendix A and previous studies (Scherer and Scherer, 2011; Krumhuber et al., 2012). The CIFERS has one more advantage: its artificial intelligent feature allows it to improve its own accuracy through data collection. It has already been trained and improved by more than 100,000 people’s emotional data before this study (see Supplementary Appendix A for more information). The CIFERS collects macro-facial expressions and micro-facial expressions, which means even when students try to conceal their emotions, the machine could still identify that emotion.

### Autonomy

Besides emotions, another factor that would affect academic persistence is autonomy support. According to self-determination theory, autonomy is the basic psychological need to make choices without pressure, external control, or compulsions (Deci and Ryan, 2000). It has been primarily acknowledged that having autonomy would support learning persistence (Pelletier et al., 2001; Vansteenkiste et al., 2004; Bonneville-Roussy et al., 2013). Specifically, in Pelletier et al. (2001) study, student-athletes who perceived more autonomy support were persistent longer in the sports that they play. Bonneville-Roussy et al. (2013) also found in a longitudinal study that college students are more persistent in learning within an autonomy-supportive environment.

Autonomy support not only associates with academic persistence but also impacts an individual’s emotional function. In Wang et al. (2007) longitudinal investigation, if under an autonomy support parenting style, children had functioned better emotionally, while a constraining parenting style would dampen children’s emotional functioning for both the United States and Chinese seventh-grade students. Another research also supported such findings. If parents and teachers showed more support for children’s autonomous behavior, children’s emotions would be more positive, and they were better at emotion regulation (Liew et al., 2011). Most of the time, autonomy support was manifested as providing choices to students (Benita et al., 2014; Lewthwaite et al., 2015). In this study, we adopted the same approach of conveying choice as a way of autonomy support.

## The Current Research

**FIGURE 1:**
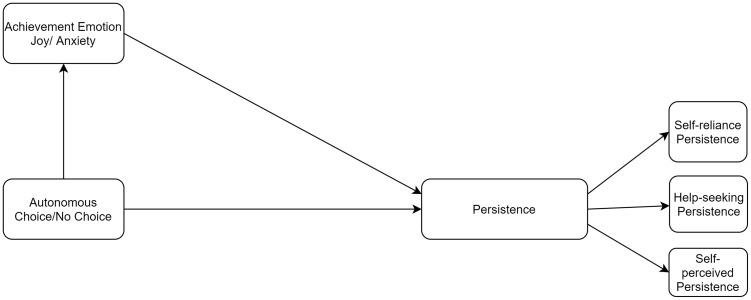
Theoretical framework.

Above all, the proposed theoretical framework of this study was presented in Figure 1. As discussed, autonomy support would induce both higher persistence and positive emotion (joy). Moreover, positive emotion could promote persistence. Many previous studies justified the association between autonomy support, emotion, and academic persistence partially. However, to our knowledge, few research studied the relationships among these three factors together, especially in an online experimental setting during a challenging time like the COVID-19 pandemic. More specifically, not many research manipulated the with/without autonomy support (choice/no choice conditions) and track students’ real-time emotions by considering different kinds of persistence (self-perceived persistence, self-reliance persistence, and help-seeking persistence).

In this study, college students were recruited online during the COVID-19 school closure. An experiment (with the control group: no choice; experiment group: choice) and online tasks were designed to record participants’ task performance, time spent on each item, and testing behaviors as academic persistence indicators. The task procedure will be specified in the “Materials and Methods” section. To address the research question stated above, four hypotheses were proposed basing on previous research.

*Hypothesis 1*: If a student’s autonomy is supported (with choice), he/she will be more persistent (both self-reliance and help-seeking persistence) comparing to students with no autonomy support (no choice).

*Hypothesis 2*: If a student’s autonomy is supported (with choice), he/she will have more positive emotion (joy) and less negative emotion (anxiety) comparing to students with no autonomy support (no choice).

*Hypothesis 3*: If a student has more intensive positive emotion (joy), then he or she will be more persistent during the task compared to the student who has less intensive positive emotion. If a student has more intensive negative emotion (anxiety), then he or she will be less persistent during the task compared to the student with less intensive negative emotion.

*Hypothesis 4*: Students who had a choice and with more intensive positive emotion (joy) should reflect a high persistence level (self-perceived, self-reliance, and help-seeking persistence). More specifically, positive emotion (joy) is expected to promote learning persistence, whereas negative emotions (anxiety) should diminish persistence. Students in the autonomy-supported (with choice) group would be more persistent and more joyful.

## Materials and Methods

### Sample and Procedures

To determine the required sample size, we conducted the Power Analysis based on the root mean squared error of approximation (RMSEA) in Structural Equation Modeling. The results showed that to achieve the power for acceptable RMSEA, the lower bound of sample sizes is 66 in each group. There were 177 college students randomly sampled from a university to ensure sufficient power for statistical inference. An experiment related to persistence was performed with participants being randomly assigned to either an experiment group (*n* = 88) or a control group (*n* = 89). In the control group, participants were allowed to choose which type of task (either math or literacy) they prefer to complete. Participants will be given no choice in the experimental group. The demographic information was presented in Table 1.

**TABLE 1:** Demographic information.

**Category**	**Number of participants**	**Percentage**
**Age**		
18–20	97	54.8%
21–22	44	24.8%
23–35	26	14.7%
36–57	10	5.6%
**Gender**		
Male	18	10.2%
Female	159	89.8%
**Race**		
White	145	81.9%
Black	28	15.8%
Asian	2	1.1%
Others	2	1%

### Instruments

Before starting the experiment, participants were asked to answer an online survey. The survey includes demographic information, a persistence scale, and an anxiety scale.

#### Persistence

The self-perceived persistence was measured by a scale developed by Howard and Crayne (2019). The scale had five items (e.g., “I keep on going when the going gets tough”), and the reliability was 0.79.

#### Controlling Factors

**FIGURE 2:**
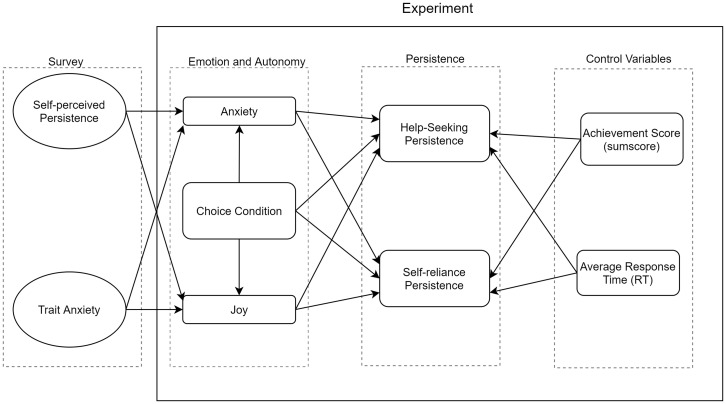
Proposed structural equation modeling analysis model.

This study also introduced several controlling factors when predicting persistence: trait anxiety (10 items; State-Trait Anxiety Inventory; Spielberger et al., 1999), gender, performance (measured by the sum scores of the task), and response time to eliminate potential confounding effects (see Figure 2).

### Experiment Procedure

The experiment aims to determine whether students exhibit different persistence levels under the choice or non-choice scenarios and different emotions. Participants had the chance to solve either math or literacy problems. Each participant was asked to answer 25 questions in both the experiment and control group. The questions were taken from the Cultural Fair Intelligence Test (CFIT) from the Genius Tests.^1^ The CFIT provides types of tasks suited to the various task conditions in this experiment.

Participants would sign up for the study through a university’s data collection system, and then the online system assigned a four-digit research ID to the student. A Zoom meeting link would be provided to the participant. The instruction would notify the participant to temporarily change their Zoom ID to their four-digit research ID temporarily before the experiment Zoom meeting. When a participant joined the research Zoom Meeting, the investigator would send him/her a consent form via the Zoom chat function. The participant would E-sign a consent form (concealed the emotion tracking information for the test’s accuracy), which indicated all the experiment information and clarified that participants could drop out of the study anytime if they feel uncomfortable. After signing the consent form, the investigator would send out an online survey (specified above) link via the Zoom chat function. When the participant finished the survey, he/she would be randomly assigned to a treatment condition (choice vs. no choice).

Participants in the experiment group could choose freely from the two groups of tasks (i.e., math and literacy tasks). Participants in the control group were presented with the math and literacy tasks, but the investigator will assign only one task type randomly to participants without providing any option for choice. Participants were informed that the task has 25 questions, and there was no time limit. Whenever they answer a question wrong, they can work on the questions by themselves more, or click the hint button, or skip the question. After assigning the group, if the participants did not have questions about the task, the investigator would be on mute and turn off the video to give the participant time to solve the 25 questions. The emotion tracking machine (specified in the next section) would be started at this point to capture participants’ facial expressions. Participants were asked to show their faces and try to face their camera the whole time.

The instructions in the assigned task stated that participants could answer as many items as possible correctly with no time limit. When participants answered a question incorrectly, they were given three options. (1) They can skip the question, in which case their answer will be considered wrong. (2) They can request a hint and then continue solving the question; if they come to the correct answer after receiving the hint, their answer will be considered correct. (3) They can continue to try to solve the question by themselves without a hint until they get the right answer. For example, if the question is, *Bruce likes 324 but not 325. He likes 2,500 but not 2,400. He likes 121, but not 122. Which does he like? (a) 900; (b) 800; (c) 700; (d) 600*. If the participant’s answer is (a), then he/she gets it correct and will automatically move to the next question. If the student’s answer is not (a), then he/she can choose to skip the question by clicking the skip button and move to the next question. The participant can also choose the hint option by clicking the hint button, and then the system will show the hint: James likes square numbers, then the student can continue to solve the problem. Persistence would be calculated according to students’ actual behaviors, and the specific method will be discussed in the “Plan of Analysis” section.

After the participant finished the task, he/she would be asked to sign a post-experiment consent form which indicated that their facial expression data were captured. If they allowed the research team to use the data, they would sign the form. After E-sign the post consent form, the participant could leave the Zoom session.

### Equipment to Measure Emotion

A facial tracking system was running through the duration of the experiment as participants complete their assigned tasks to track their emotional state. The Contain Intelligent Facial Expression Recognition System (CIFERS) camera would capture participants’ real-time facial expressions from the Zoom window. CIFERS is a software program that uses macro- and micro-expression data modeling to track individuals’ facial movements and determine participants’ cognitive and psychological states. The software recorded participants’ 11 basic emotions and emotional changes over time (more information about the equipment appears in Supplementary Appendix A). This study used joy and anxiety as the targeted emotions.

### Plan of Analysis

Data analysis was conducted following two steps. In the first step, we categorized each item responses for each participant into three types of persistence behaviors (self-reliance behavior, help-seeking behavior, and low persistence) according to three criteria: (1) the number of times participants click the “submit” button (participants have to try at least one); (2) whether they clicked “hint” button, and (3) whether they skipped an item. Moreover, the total response time for each group of items was recorded (*T*_*sr*_, *T*_*hs*_, *T*_*l*_) which represented the time length they showed self-reliance behavior, help-seeking behavior, and low persistence accordingly during the experiment. In the second step, we analyzed five regression models in which the three types of persistence were considered as outcomes.

As shown in Table 2, each participant’s persistence was defined using participants’ behaviors. Specifically, the criteria for item-level persistence were as follows: items with self-reliance behavior – the individual responded to an item more than once, did not ask for hints, and did not skip the item; items with help-seeking behavior – similar to self-reliance persistence except the individual asked for hints; items without persistent behavior – the individual answer incorrectly and skipped the items, so they did not demonstrate high or moderate persistence. The persistence index Yp in this study was defined as the total time spent on the items which show specific persistence behavior for participant p. We set J as the total number of items, p as the person index, and i as the item index. For example, each participant’s time interval showing self-reliance behavior can be computed as follow:


(1) $$Y_{p}=\sum_{j=1}^{J}\text{​}T_{p}{}_{j}I_{j}$$

where *Y*_*p*_ is a *P × 3* test-level persistence score vector representing three types of persistence scores for total participants. *I*_*j*_ is an indicator matrix for item j suggesting whether this item shows specific persistence behavior, whose values were either 0 or 1 and *I* is *J × 3* matrix; for example, *I*_1p_ = [1, 0, 0] indicated that Item 1 shows self-reliance behavior for person p. *T*_*pj*_ represents the time response vector for item *j* answered by person p and then *T* is a *P × J × 3* matrix.

**TABLE 2:** Scoring rules for persistence.

**Persistence**	**HINT**	**SKIP**	**TRY**
Self-reliance	N	N	>1
Help-seeking	Y	Y/N	>1
No persistence	Y	N	1
No persistence	N	Y	1
No persistence	Y	Y	1

We use the regression model below to examine Hypothesis 1:


(2) $$Y_{\mathrm{persis}}=\mathrm{SS}+\mathrm{RT}+\mathrm{choice}$$

where *Y*_*persis*_ is one of two types of persistence indices of interest (help-seeking persistence or self-reliance persistence), SS (sum scores) represents the achievement scores which were computed with the number of items each participant answer correctly, RT (response time) represents the total time each participant used, choice indicates whether the participant was allowed to select the type of task.

To examine Hypothesis 2, we estimated the following regression models:


(3) Anxiety=choice+gender+trait⁢_⁢of⁢_⁢anxiety

(4) Joy=choice+gender+trait⁢_⁢of⁢_⁢anxiety

where *Anxiety* and *Joy* are the maximum level of that emotion of participants during the experiment; *gender* indicates whether the participants are females or males; *trait_of_anxiety* indicates the state of anxiety level measured by State-Trait Anxiety Inventory. There were two major reasons for using the maximum level of anxiety and joy. Firstly, according to previous literature, only a higher level of emotional arousal would induce behavior and influence the decision-making process (Kaufman, 1999; Hu et al., 2015). Secondly, using other indices, for instance, mean (total emotion show/time), would be inaccurate since students were not showing joy or anxiety all the time during the experiment.

To examine Hypothesis 3, we used the regression model as follow:


(5) $$Y_{\mathrm{persis}}=\mathrm{SS}+\mathrm{RT}+\mathrm{Anxiety}$$

(6) $$Y_{\mathrm{persis}}=\mathrm{SS}+\mathrm{RT}+\mathrm{Joy}$$

Finally, to examine Hypothesis 4, a structural equation model containing all variables was fitted to test the effects of emotion and choice condition (see Figure 2). In SEM, the dependent variable was either the emotion index (*Anxiety* or *Joy*) or persistence index (*Y*_*persis*_); independent variables were the choice condition, anxiety, and joy. Other controlling variables included anxiety traits, achievement scores (SS), and average response time (RT). To examine the goodness-of-fit of SEM, root mean square error of approximation (RMSEA), and maximum likelihood (ML)-based standardized root mean squared residual (SRMR) were reported to evaluate the adequacy of the model. RMSEA and SRMR values close to or lower than 0.08 are acceptable, although values approaching 0.05 are preferable (Hu and Bentler, 1999).

## Results

Table 2 showed participants’ demographic information, including age, gender, ethnicity, and sexual orientation. Table 3 showed the descriptive statistics of dependent and independent variables, including the persistence scores, the proportion of choice condition, maximum joy, and anxiety level. The ranges for persistence scores, joy, and anxiety are all 0–100. The emotion data were extracted from the CIFERS background data.

**TABLE 3:** Descriptive statistics.

**Variables**	**Mean or %**	**SD**
Help-seeking persistence	264.96	248.53
Self-reliance persistence	180.53	161.82
Participant in choice condition	49.7%	0.50
Maximum joy level	61.99	45.39
Maximum anxiety level	97.69	6.15

For *Hypothesis 1*, controlling the effects of response time and sum scores (task performance), we found that whether having a choice (autonomy support) has significant positive effect on self-reliance persistence (β = 0.290, *p* < 0.05) but no significant effect on either help-seeking persistence (β = −0.064, *p* = 0.587) or self-perceived persistence (β = 0.010, *p* = 0.912). The results partially support Hypothesis 1 that if a student’s autonomy is supported, he/she may have higher self-reliance persistence.

For *Hypothesis 2*, the results showed that after controlling the effects of the anxiety trait and gender, there is no significant relationship between participants’ maximum joy level with whether they have choice or not (β = 8.765, *p* = 0.205). There is also no significant association between participants’ maximum anxiety level with whether they have a choice (β = −0.806, *p* = 0.400). Thus, the results did not support Hypothesis 2.

For *Hypothesis 3*, the regression results showed that participants’ maximum level of joy had an approximately significant association with self-perceived persistence at the level 0.05 (β = 0.002, *p* = 0.066), but we did not confirm that it related to the help-seeking (β = −0.002, *p* = 0.210) and self-reliance persistence (β = −0.002, *p* = 0.347). On the other hand, participants’ maximum anxiety level seemed to have a significant negative association with self-reliance persistence (β = −0.025, *p* < 0.05) but no significant relationship with the other help-seeking persistence (β = 0.009, *p* = 0.306). Thus, the results partially support Hypothesis 3.

**FIGURE 3:**
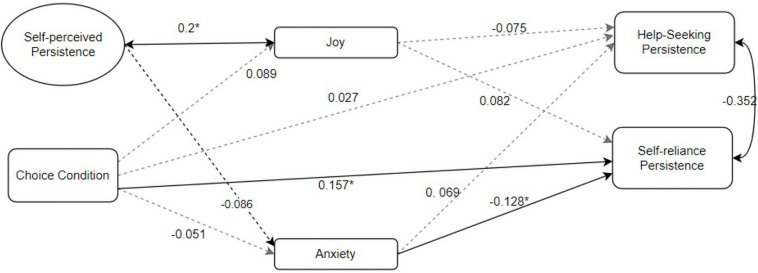
Structural equational modeling results. *p < 0.05. Omitted controlling factors include: gender (help-seeking persistence*: β = 0.107, *p* = 0.049; self-reliance persistence: β = –0.103, *p* = 0.055; anxiety: β = 0.003, *p* = 0.968; joy: β = –0.071, *p* = 0.339); trait of anxiety (help-seeking persistence: β = –0.026, *p* = 0.668; self-reliance persistence: β = –0.073, *p* = 0.356; anxiety: β = 0.079, *p* = 0.353; joy: β = –0.030, *p* = 0.722); sum scores (help-seeking persistence*: β = –0.122, *p* = 0.025; self-reliance persistence*: β = 0.349, *p* < 0.001); Response Time (help-seeking persistence*: β = 0.693, *p* < 0.001; self-reliance persistence: β = 0.069, *p* = 0.307).

For *Hypothesis 4*, as shown in Figure 3, the SEM has acceptable model fit [SRMR = 0.072, RMSEA = 0.049, CFI = 0.918, TLI = 0.9, χ^2^ (203) = 284.57, *p* < 0.01]. The results of SEM with maximum likelihood (ML) estimation showed that controlling for the effects of sum scores (task performance), response time, gender, and anxiety trait, whether having a choice (autonomy support) was significantly associated with self-reliance persistence (β = 0.157, *p* = 0.022) but not significantly related to the help-seeking persistence (β = −0.027, *p* = 0.62), which partially supports our Hypothesis 4. As for the effects of emotion on persistence, the results showed that the maximum anxiety level has an approximately significant effect on self-reliance persistence at the alpha level 0.05 (β = −0.128, *p* = 0.060) but not on help-seeking persistence (β = 0.069, *p* = 0.2). Additionally, self-perceived persistence significantly affects the maximum joy level (β = 0.2, *p* < 0.001).

## Discussion

This study explored the association among academic persistence, autonomy support, and emotions within the online environment during the COVID-19 pandemic. Results of regression and structural equation models supported some of the hypotheses.

Partially consistent with Hypothesis 1, having choice did promote students’ self-reliance persistence but did not show relation with help-seeking persistence. Hypothesis 2 was not supported by our study, as different choice conditions did not influence students’ joy or anxiety. Hypothesis 3 was partially supported, as being joyful during the task will promote students’ self-reliance persistence, and being anxious during the task would diminish self-reliance persistence. Moreover, emotions did not relate to other kinds of persistence. After controlling for participants’ trait anxiety, gender, response time, and sum scores (task performance), the Hypothesis 4 testing results showed that having choice was positively associated with self-reliance persistence. In contrast, no choice and having high anxiety were related to lower self-reliance persistence. Moreover, if the self-perceived persistence is high, then the participants were more likely to have high intensity of joy during the task.

Additionally, the relationships among different kinds of persistence were justified in this study. Self-reliance persistence and help-seeking persistence were associated with each other negatively, while self-perceived persistence did not show any significant association with either self-reliance persistence or help-seeking persistence. Such results indicated that how much a student believed he/she is persistent does not represent how he/she would actually behave during the problem-solving process. Moreover, students who adopted self-reliance persistent would be less likely to adopt help-seeking persistence. Specifically, if an individual usually solves problems by himself/herself would be less likely to seek help from others, and vice versa, but eventually, people would achieve their goals.

Findings of the association between choice and academic persistence were consistent with previous research and justified the direct relationships of choice with different types of persistence (Bonneville-Roussy et al., 2013; Yurdakul, 2017). After controlling for task performance, trait anxiety, performance, and response time, college students in the choice group would show more self-reliance persistence in the problem-solving tasks. This finding means no matter he/she good at math or not, by having choice, students were more likely to solve challenging tasks by themselves. However, whether students would like to seek help to continue solving the questions did not show a significant relationship with having or not having a choice. Such finding showed that given a choice or not did not affect a students’ likelihood to seek help to continue solving a challenging task.

Findings of the association between emotions and academic persistence were partially aligned with previous studies and also added new perspectives to current literature. Positive emotion (joy) promoted self-reliance persistence, while negative emotion (anxiety) undermines self-reliance persistence (Tulis and Ainley, 2011; Yu et al., 2020). Specifically, indicated by the real-time emotional tracking system, students who experienced joy would be more likely to solve the problem by themselves, but if students experience high intensity of anxiety, they would not continue working on the problem. Interestingly, when participants’ self-perceived persistence was high, they would show more joy during the problem-solving process. However, help-seeking persistence did not show any association with both joy and anxiety.

There are several theoretical contributions of this research by using an experimental approach and tracking real-time emotions. Firstly, this study offered a new approach to define academic persistence. To the best of our knowledge, this study is the first one that considered academic persistence from both trait and state perspectives. It conceptualized academic persistence from three aspects: self-perceived persistence (trait), self-reliance persistence (state), and help-seeking persistence (state). As suggestions by our research, individuals have their perceived persistence, but they could behave differently in different situations, so self-perceived (self-reported) persistence might not be reliable in certain circumstances. Whether an individual good at a learning activity or not, his or her perceived persistence does not lead to more persistent behaviors in that activity.

Secondly, this study provided new ways to measure academic persistence. Going beyond previous studies which used the time or frequency to measure persistence, this study used individuals’ actual persistent behaviors as the indicators. This behavioral tracking approach could provide a new perspective to study multidimensional and dynamic cognitive and metacognitive processes similar to persistence, such as self-regulation, critical thinking, or creativity.

Thirdly, adding on previous research which addressed emotions (Linnenbrink and Pintrich, 2002; Tulis and Ainley, 2011) and autonomy support (Pelletier et al., 2001; Vansteenkiste et al., 2004) could promote academic persistence, the current study specified that only self-reliance persistence would be influenced by emotions and autonomy support. If students decided to ask for help to overcome a difficulty, their persistence would not be influenced by emotions and autonomy support. Contradicting previous research (Wang et al., 2007; Liew et al., 2011), as a way of autonomy support, having choice or not seemed uninfluential toward college students’ emotions in an online setting. However, more research is needed to examine such finding. Fourthly, instead of using self-report data, this study is one of the first studies that considered real-time emotions and the intensity of emotions during students’ problem-solving process.

Besides theoretical contributions, the present findings have several practical implications. Firstly, if students are going through a difficult time (e.g., life tragedies, the COVID-19 pandemic), a teacher should provide more choices for students to help cultivate students’ self-reliance learning persistence in an online learning environment. Secondly, if self-reliance persistence is not always achievable, teachers should be more assessable in ways like instant feedback or prompt email reply to promote students’ help-seeking persistence. Thirdly, providing emotional support would help with students’ self-reliance persistence. The proper way of emotional support would significantly improve students’ self-reliance persistence. Recent research indicated that teachers’ emotions and teacher-student relationship could impact students’ emotions (Goetz et al., 2021), so teachers being positive and cheerful would promote students’ positive emotion, and eventually promote self-reliance persistence. Fourthly, this research would be helpful for establishing a more supportive and sustainable online learning environment by incorporating more autonomy supports and instant feedback system in the course structure or teacher-student communications. Such environment would promote students’ self-reliance and help-seeking persistence, which could potentially alleviate learning loss, drop-out, and learning anxiety during difficult life period (e.g., COVID-19, grief, mental health problems, or other life tragedies).

## Limitation and Future Directions

This study provided both new theoretical and practical contributions, but some findings should be interpreted with caution due to the following limitations. Firstly, the majority of our participants were female college students, so the generalizability to male students might be wakened. Another limitation of this study is that only one summative score of emotions (maximum level) was used in the analysis. Action unites (i.e., the response process data), a novice approach to explore assessment data, were not employed in this study. Thus, the dynamic process of participants’ emotions was not considered in this study. The association between fluctuated emotions and persistence behavior has not been examined in the study because SEM cannot analyze time-series data. Moreover, other academic emotions, such as frustration or boredom, should be considered essential for learning persistence (D’Errico et al., 2018; Narayan and Sharma, 2021). Future studies should address these emotions.

In future studies, dynamic structure equation models (DSEM) could address the causal relationship between emotion and persistence. Future studies could also include more diverse student samples, which could improve the findings’ generalizability (e.g., including samples from other countries). A more racially and ethnically diverse population will have more practical implications, which can apply to different learning environments with different cultural backgrounds. Additionally, longitudinal analysis of the association between emotion and persistence is needed to infer the potential causal relationship between emotion and persistence. Further consideration of other emotions and see students’ facial signals as convey evaluative (students’ criticism or disagreement) meanings that can contribute to understanding on the other side students’ autonomy (Poggi et al., 2013).

Regardless of the limitation stated above, this study provided meaningful results on how real-time emotions and autonomy support influence different kinds of academic persistence with an experimental design and a new method of assessing emotions.

## Data Availability Statement

Raw data were generated at the Micro-Facial Expression Tracking Lab, University of Alabama. Derived data supporting the findings of this study are available from the corresponding author YW on request.

## Ethics Statement

The studies involving human participants were reviewed and approved by The University of Alabama’s Institutional Review Board. The patients/participants provided their written informed consent to participate in this study.

## Author Contributions

YW designed the research and drafted the manuscript. JZ was in charge of the data analysis. HL contributed to the literature review and data collection. All authors contributed to the article and approved the submitted version.

## Conflict of Interest

The authors declare that the research was conducted in the absence of any commercial or financial relationships that could be construed as a potential conflict of interest.

## Publisher’s Note

All claims expressed in this article are solely those of the authors and do not necessarily represent those of their affiliated organizations, or those of the publisher, the editors and the reviewers. Any product that may be evaluated in this article, or claim that may be made by its manufacturer, is not guaranteed or endorsed by the publisher.

## Footnotes

1
https://geniustests.com/iq-tests/culture-fair-intelligence-scale
