# Supplementary material for: An Online Experiment During COVID-19: Testing the Influences of Autonomy Support Toward Emotions and Academic Persistence
Source: Front Psychol. 2021 Oct 11;12:747209. doi: 10.3389/fpsyg.2021.747209 (PMC8542910; doi:10.3389/fpsyg.2021.747209)
Supplement: Supplementary file 1 [file Data_Sheet_1.PDF]

Appendix A. CIFERS Information V 2.01

Product Overview

The Intelligent Micro-Facial Expression Recognition System is a software that built based on Ekman & Friesen's (1978; 2003) Facial Action Coding System. The system has been tested and adapted to more than 10,000 users in China. The software could recognize an individual's psychological activities in real-time. It combines computer vision processing technology with micro-expression and provides a judgment system to model and learn large numbers of cases through artificial intelligence platform.

Through the offline training of terabyte data, the system obtains micro-expression models corresponding to different cognitive emotion, especially the micro-expression data modeling. The AI-assisted with cognitive brain recognition using joint semi- supervised learning to establish the deep learning training model, forming a complete micro-expression semantic tag.

The system stimulates the psychological transient characteristics through external stimulation such as vision and hearing to obtain facial muscle micro-changes. It can make data fusion through the movement trend of facial muscle and static combination to construct the auxiliary judgment system basing on facial micro-features.

Application Scenario

Real-time interview and assisted judgment:

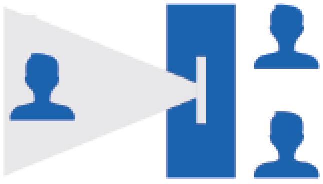

Continuously capture the person's facial activity during the real-time

interview process and give a certain alarm when specific micro-expression combinations appear.

Static video-assisted analysis:

Observe and analyze the recorded video material to confirm whether the person's psychological state.

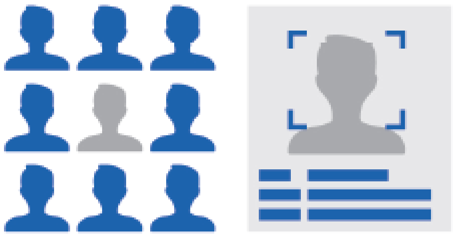

Product Features

- ✓ Non-contact

  The traditional interview research and judgment system generally use contact-type wearable devices to obtain the tested person's specific physiological indicators. However, this system uses a non-contact method to identify facial micro- expression by using computer and image analysis and deep neural network learning methods.

- ✓ Real-time

  The system can provide real-time prediction suggestions through the non-contact high-definition camera and micro-expression test system.

- ✓ Universality

  This system highly summarizes the expert's professional experience and applies computer learning to the system through computer technology, which can be easily operated by ordinary case handlers.

- ✓ Easy to use

  Neither sophisticated contact devices need to be connected to the participated subjects, nor complex software operations and professional

knowledge need to be used in performing this system.

- ✓ High accuracy recognition ability

  Based on deep learning, a highly abstract micro-expression facial model is studied in terabyte data.

- ✓ Flexible deployment

  Deployed in a non-hierarchical architecture, no need for complex environment deployments, and stand-alone available.

Software Function

- Built-in offline micro-expression recognition engine, this system can capture and identify key facial micro-expressions with no need to connect to

  the Internet.

- Built-in micro-expression library trained by terabyte level massive data offline, it can accurately locate and analyze the tested person's key expression emotions during the interview or teaching process.

- Supporting real-time video stream analysis, such as RTSP stream of network camera standard, external USB camera, and host with a built-in camera.

- Supporting video file import in multiple formats, it can analyze the micro-expressions emotion of post-mortem interview in the past process imported

  by the video file.

- During the process of interview or teaching scenario or video playback, the micro-expression emotion indicators can be displayed in a branch

curve manner in real-time;

- 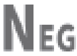 Multi-dimensional psychological and emotional indicators display: This system provides NEG, DCP, ANX, DNG display of various emotional indicators, which can be used to assist the case-handling personnel in different dimensions to accurately research and judge;

- 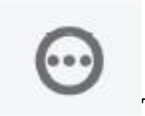 The intelligent judgment of emotional analysis results during the process of an interview or video playback: The system displays the results of intelligent judgments in real-time according to the emotional, psychological indicators and the adaptive background algorithm;

Technical Indicators Accuracy

Facial expressions capture accuracy:
≤50ms; Number of face coding units: 47;
Emotional resolution: 7 basic emotions, 4 kinds of extended emotions; Video file resolution: The imported video file is up to 1920*1080;
Video file playback rate: ≥24fps;
Video formats supported: MPEG (PS, TS), AVI, ASF, WMV, MP4;

System Requirements

Hardware requirements:
Processors of Intel I7-8700 and above, Memory of 8GB and above, resolution of 1920x1080 and above, video capture device (USB camera or network camera) Software requirements:
Operating systems of Windows 7 and above (Windows 10 is recommended), Versions of Dot Net Framework 4.6 and above, VC operating environment of 14.0 and above.

Analysis Display

Including more than ten basic emotions: e.g., happiness, attention, sadness, anger, fear, disgust, deny, ignorance, anxiety. There are multiple indicators of emotion:

Emotion intensity:

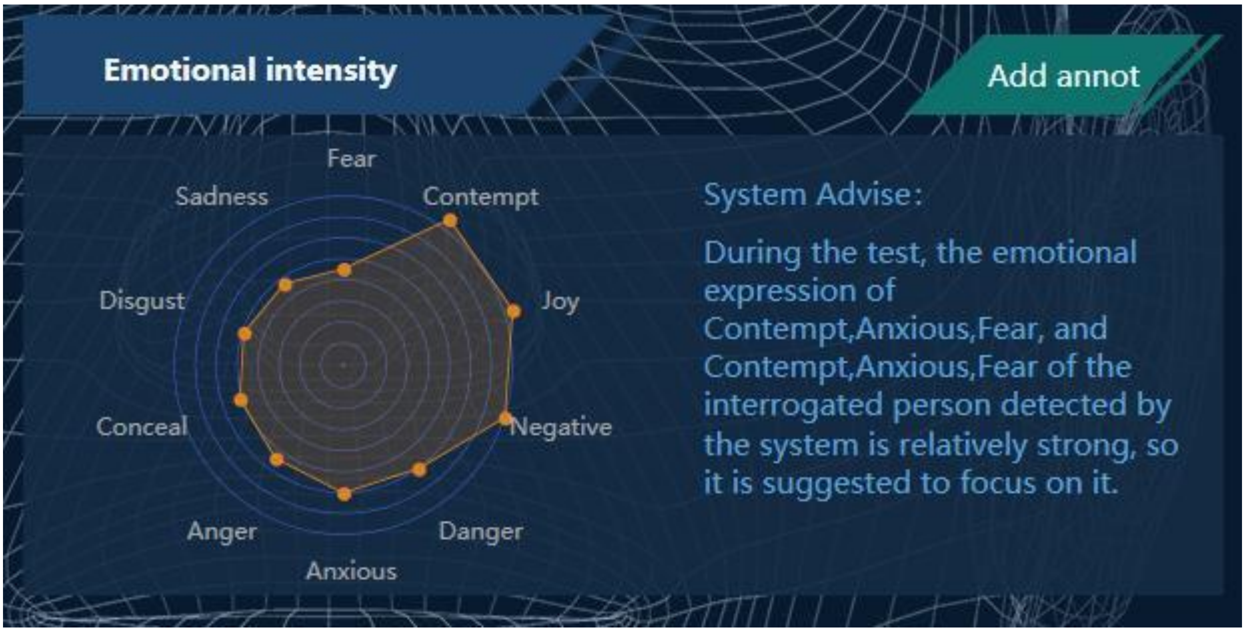

Emotion Distribution:

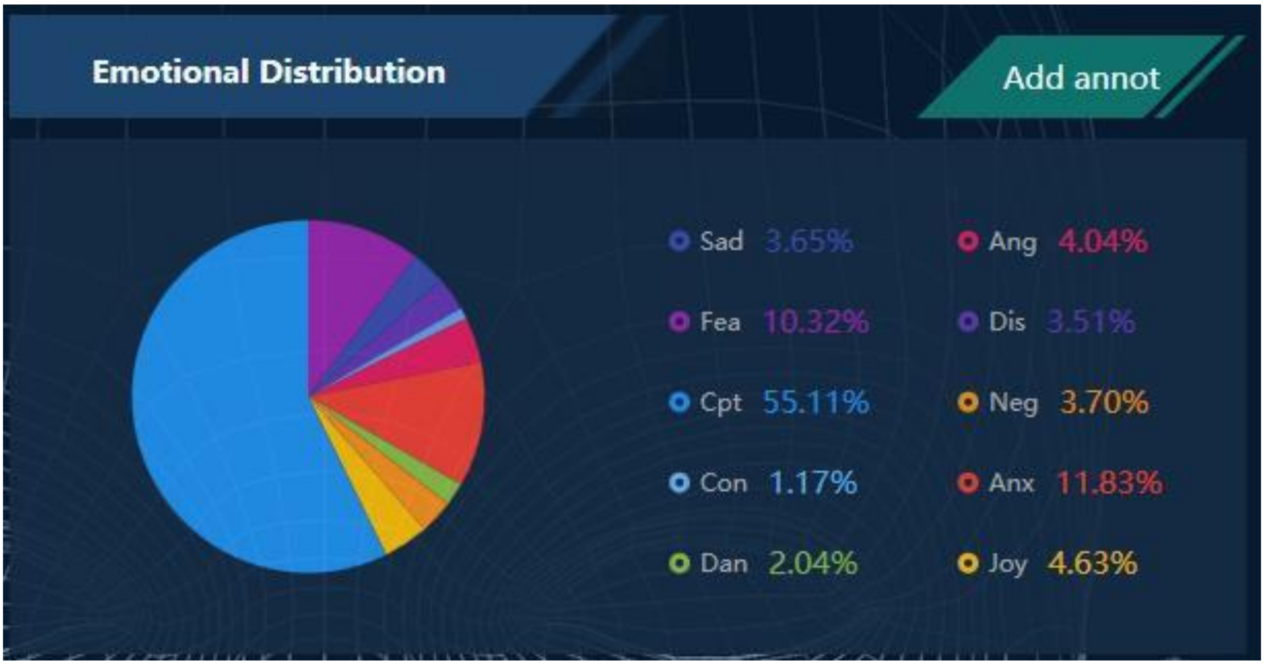

Track Emotion Change Overtime:

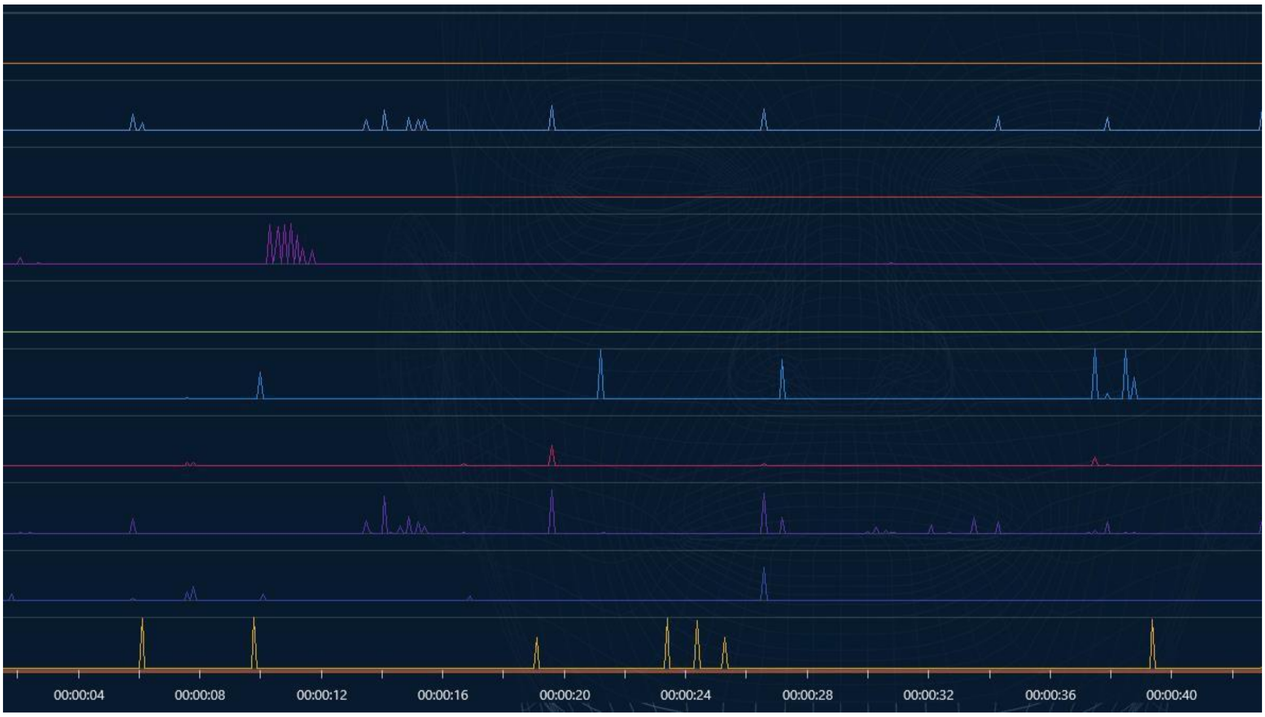

Actual Product

It is the size of two regular computers that bundled up.

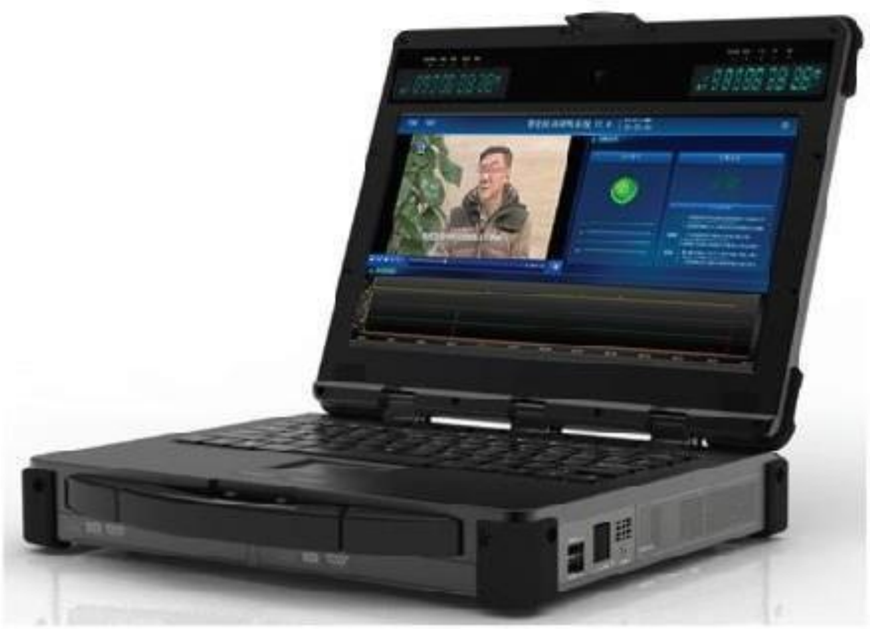

Reference:

Ekman, P., & Friesen, W. V. (1978). *Manual for the facial action coding system*. Palo Alto: Consulting Psychologists Press.

Ekman, P., & Friesen, W. V. (2003). *Unmasking the face: A guide to recognizing emotions from facial clues*. Ishk.

[i]

[i] Note: For more information, please go to http://www.contain.com.cn/
